# Friction-Wear and Noise Characteristics of Friction Disks with Circular Texture

**DOI:** 10.3390/ma17102337

**Published:** 2024-05-14

**Authors:** Biao Ma, Weichen Lu, Liang Yu, Cenbo Xiong, Guoqiang Dang, Xiaobo Chen

**Affiliations:** Beijing Institute of Technology, Beijing 100081, China; mabiao@bit.edu.cn (B.M.); 3120210312@bit.edu.cn (W.L.); xiongcenbo@bit.edu.cn (C.X.); dangguoqiang223@163.com (G.D.); 3120205194@bit.edu.cn (X.C.)

**Keywords:** texture, caliper disc brake, wear, noise, growth rate

## Abstract

The reduction of friction-induced noise is a crucial research area for enhancing vehicle comfort, and this paper proposes a method based on circular pit texture to achieve this goal. We conducted a long-term sliding friction test using a pin-on-disc friction and a wear test bench to verify the validity of this method. To compare the friction noise of different surfaces, texture units with varying line densities were machined on the surface of friction disk samples. The resulting friction-wear and noise characteristics of the samples were analyzed in conjunction with the microscopic morphology of the worn surfaces. The results indicate that surfaces with textures can delay the onset of squeal noise, and the pattern of its development differs from that of smooth surfaces. The noise reduction effect is most evident due to the proper distribution of textures that can form furrow-like wear marks at the wear interface. The finite element results demonstrate that this morphology can improve pressure distribution at the leading point and reduce the tendency of system instability.

## 1. Introduction

Friction brakes are an important component that rely on friction to generate braking torque and can have a significant impact on driving safety. Among them, the caliper disc brake has simple structure and convenient maintenance, and is widely used in vehicles and engineering machinery equipment [1,2]. However, along with the wear of brake pads, high-frequency vibration and noise will be generated, which seriously affects the comfort of the passenger. Therefore, reducing the brake noise under the premise of ensuring the braking performance of the vehicle is still an important target of vehicle design [3,4,5]. Research into frictional vibration noise dates back to the 1930s, but due to the complexity of frictional behavior and the random nature of vibration noise, there is still no uniform understanding of the mechanism of frictional noise generation and effective suppression methods. A widely accepted theory is that the friction squeal of brakes mainly originates from self-excited vibration, and this behavior is influenced by the surface contact pressure distribution [6]. However, there is still a lack of research on the relationship between surface morphology and friction-induced vibration and noise [7,8].

Recent studies indicate that textured surfaces with non-smooth morphology exhibit excellent performance in noise reduction and wear resistance [9,10]. To optimize the texture structure, numerous experimental studies have been carried out on textured surfaces with varying parameters, such as texture shape and size [11,12]. Zhang et al. investigated the frictional wear of grooved textures under sand-containing oil lubrication conditions. The study showed that the grooves were able to capture grit, preventing surface scratches and improving wear resistance [13]. Babu et al. studied the frictional characteristics of textures of different shapes and densities under different lubrication conditions, and verified the effect of the texture on the frictional characteristics using finite element simulations [14]. Xie et al. conducted friction tests on steel surfaces prepared with triangular textures. The results showed that the oil in the texture units could ensure the continuity of the oil film at the friction contact interface, and provide anti-wear effects [15]. Arslan et al. compared the effect of pit textures of different diameters and depths on the frictional properties of amorphous hydrogenated diamond-like carbon (DLC), and showed that only specific sizes of texture units could improve the frictional performance [16].

There has also been a great deal of research into the noise reduction effects of texture [17]. Wang et al. conducted an experimental study on the noise reduction effect of pit texture in tapered roller bearings under under-lubricated conditions. The results indicated that bearings with pit textures have good potential for vibration absorption and noise reduction compared with smooth surfaces [18]. Liu et al. investigated the effects of different surface textures on stainless steel samples. Their friction and wear tests demonstrated that the textures can reduce the coefficient of friction (COF), increase wear resistance, and improve dynamic properties. Additionally, surface textures can reduce friction-induced vibration and noise by up to 95% and 66%, respectively [19]. Chen et al. analyzed the relationship between the friction vibration attractor and the COF by combining chaos theory. They concluded that the existence of the pit unit can effectively improve friction performance and inhibit friction vibration [20]. Xue et al. created a composite surface on GCr15 steel by adding grooves and Sn–Ag–Cu. They discovered that this combination increased the amplitude of friction fluctuation, resulting in a wear surface with noticeable wrinkles. Additionally, the friction-induced vibration and noise were suppressed [21]. Li et al. investigated the effect of different textures on the squealing tendency of brakes using complex eigenvalue analysis, and the results showed that brakes with texture characteristics had a lower tendency to vibrate [22]. Zhou et al. added a solid lubricant to the texture and found that the presence of the texture could improve the pressure distribution, which was conducive to achieving a stable friction coefficient. By controlling the friction coefficient of the system to suppress the unstable vibration, the friction noise can be effectively reduced [23].

Copper-based friction materials have the advantages of high wear resistance and high thermal conductivity, but are also prone to high intensity friction noise [24,25,26]. However, there are still few studies on the noise reduction of this material. In this paper, a friction noise suppression method based on circular pit texture is proposed, and the noise reduction mechanism of the method is explained from the perspective of surface morphology—coefficient of friction—vibration noise. Further pin-on-disk tests of samples with different texture densities are carried out to validate the effectiveness of the noise reduction method. The results show that the presence of textures can reduce the wear at the friction interface and reduce the friction noise, which provides a new method for vibration and noise reduction in friction braking systems in engineering practice.

## 2. Materials and Methods

### 2.1. Test Equipment

**Figure 1:**
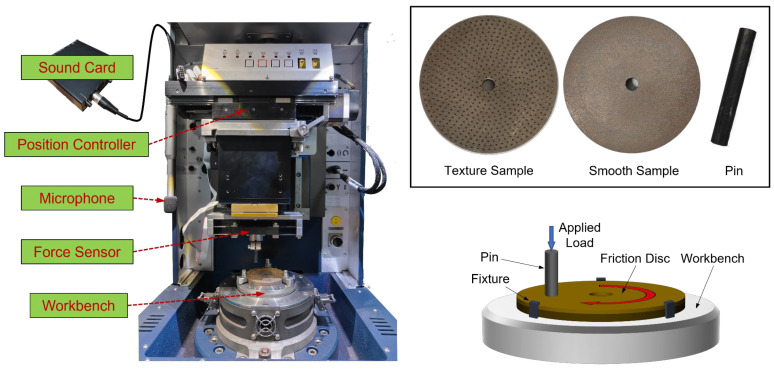
Test equipment and schematic diagram.

The main equipment used in this study include a multifunctional friction and wear test bench (UMT-5 produced by Bruker, sourced in Beijing, China), a Behringer ECM8000 test microphone, a Roland Rubix22 sound card, and a white light interferometer. The arrangement of these equipment and the schematic diagram of the friction system are shown in Figure 1.

Most importantly, the pin-on-disk dry contact test module is employed in the multifunctional friction and wear test bench, which can be divided into upper and lower assemblies. The upper assembly includes a position controller, a force sensor, and a steel pin, while the lower assembly consists of a friction disc and a workbench. The pin is fixed under the force sensor and remains stationary during the test, with the normal pressure applied by the position controller. The friction disc is fixed to the workbench by fixtures and is rotated by a motor underneath. At the beginning of the test, the pin is driven downward by the position motor to establish contact with the friction disc, and then the pressure is applied by the tiny movement of the motor. When the error between the actual pressure and the set pressure is less than 1% for five seconds, the workbench and the friction disc start to rotate by the rotary motor. This test bench is capable of acquiring signals of force, displacement, and rotational speed signals during the test and calculating coefficients of friction (COF) in real time at a sampling rate of 100 Hz. Moreover, the microphone and sound card are used to capture friction-induced noise at a sampling rate of 44.1 kHz. The sampling point is located inside the protective cover, which can effectively isolate external noise.

The white light interferometer produced by Bruker is capable of scanning the micro-morphology of the sample surface in a non-contact manner, avoiding damage to the sample surface and contributing to the analysis of the friction and wear characteristics of different textured surfaces. The surface morphology of the worn area on the friction disc is obtained at the end of the friction test. By comparing wear marks of the samples, the influence of texture on friction and wear characteristics is assessed. Differences in surface morphology can alter the contact state of the friction pair, and understanding the wear mechanism of different surfaces can aid in interpreting changes in COF and noise.

### 2.2. Test Samples

**Figure 2:**
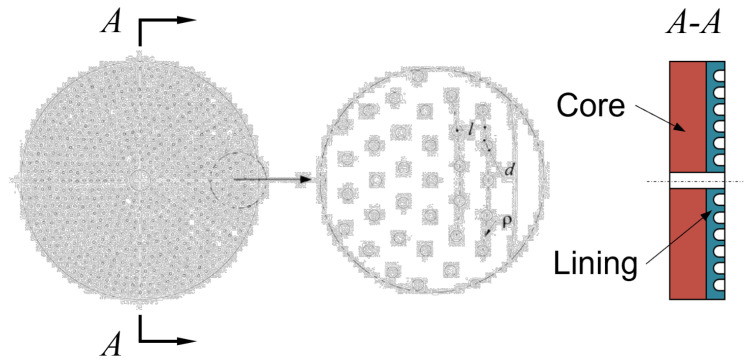
Friction disc structure.

Figure 2 shows the friction disc sample used in the test, which consists of a core plate made of 65 Mn steel and a friction lining sintered through a copper-based powder metallurgy process. The thickness of the core plate and the friction lining is 4 mm and 2 mm, respectively, and the outer diameter of the friction disc is 68 mm. Copper-based powder metallurgy materials usually form friction pairs with 65 Mn steel, so the pin is also made of 65 Mn steel with a diameter of 6 mm and a length of 40 mm. The material parameters of the test samples are given in Table 1.

As the circular micro-pit texture offers improved friction performance and is easy to process, this study adopts a laser processing technology to add the circular texture features on the friction lining surface. The texture feature of the friction lining surface is machined using laser machining technology, and the circular micro-pit type texture with good friction properties and easy machining is selected in this paper. To be more specific, the pit units have a diameter of *d* = 0.8 mm and a depth of *h* = 0.4 mm, arranged in a concentric circumferential distribution with a radial distance *l* = 2 mm between adjacent circumferences. To ensure an objective evaluation of the sparsity of texture units, we define line density as the number of texture units per unit length, calculated as follows:

(1) $$\rho =\frac{N_{t}}{2\pi r}$$

where *N_t_* is the number of texture units on a given circumference, and *r* is the radius of that circumference. The comparison of friction and noise characteristics is carried out under three textures of the smooth surface (ρ = 0), ρ = 0.3, and ρ = 0.5, and the corresponding sample serial numbers are denoted by T-R, where R represents its texture unit line density ρ = R/10. For most calliper disc brakes, the maximum contact pressure of the brake is approximately 3 MPa. Based on the principle of similarity, the operating conditions of the automotive calliper disc brake under low speed and heavy load are simulated by ensuring that the test system and the brake work under the same contact pressure and linear velocity. The test parameters are shown in Table 2, and the duration of each test was 10 min.

### 2.3. Growth Rate Extraction

**Figure 3:**
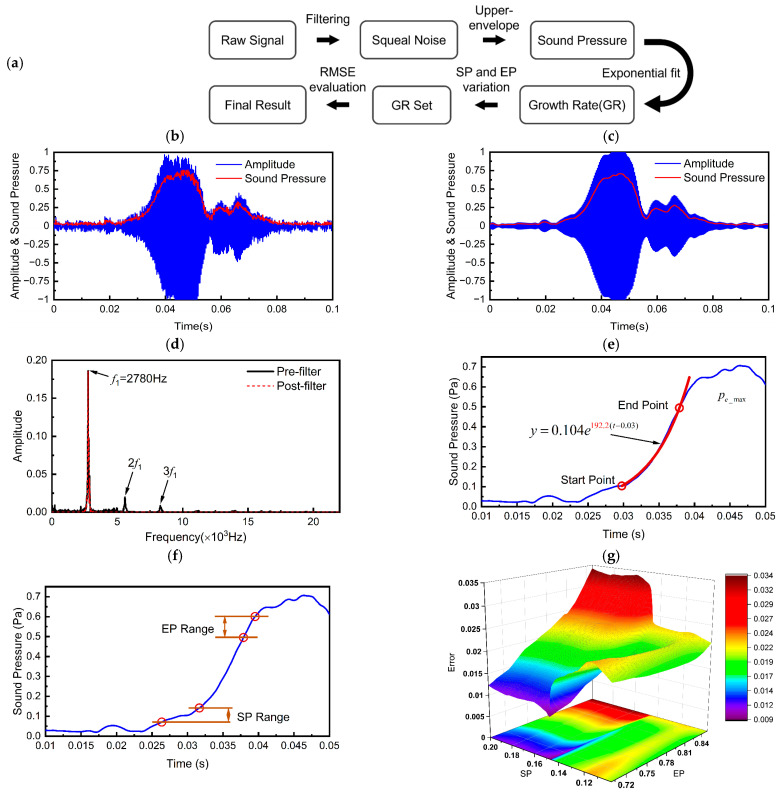
Methodology for calculating growth rates: (**a**) Signal processing procedure; (**b**) Raw signal; (**c**) Filtered signal; (**d**) Comparison in frequency domain before and after filter; (**e**) Exponential fitting for growth rates; (**f**) Start and end point variation intervals; (**g**) RMSE set.

Taking a typical noise signal during friction of sample T-0 as an example, the method of extracting the noise growth rate (GR) from the transient time series signal is introduced, and the extraction process is shown in Figure 3. Firstly, the raw signal is filtered to separate the sharp high-frequency signal that appears in the later stage of the test, and then the signal is divided into frames to calculate the sound pressure of the noise signal. The growth phase of the sound pressure is fitted with an exponential function, and the corresponding exponent is the required noise growth rate. Since the variation of the start and end points will have a significant effect on the fitting results, the root-mean-square error within the fitting interval is adapted to evaluate the fitting effect, and the fitting exponent with the smallest error is selected as the final value of the noise growth rate.

#### 2.3.1. Raw Signal Filtering

The raw time-domain signal acquired contains a lot of white noise signals, thus, using the original signal to fit the noise growth rate directly will affect the accuracy of the results. In this long-term sliding test, the acoustic emission (AE) showed periodic characteristics related to the rotational speed, and low frequency oscillations with small amplitudes could be observed between adjacent acoustic emission events. Although the sound pressure in the later stage of the test was mainly determined by high frequency signals, these low frequency signals can still significantly affect the selection of the fitting start and end points, which reduces the consistency of the fitting results at different stages of the test. Therefore, it is necessary to filter the raw signal before extracting the growth rate of the acoustic emission signal.

The frequency of the squeal noise is located by performing a fast Fourier transform (FFT) on the raw signal and identifying the center frequency of the filter. The passband interval is the region with a width of ∆f = 200 Hz on either side of the center frequency. To obtain high frequency components that are highly correlated with the acoustic emission phenomena, the raw signal is digitally filtered using a Blackman window in zero phase. It can be seen from Figure 3d that the high-frequency noise in the friction process originates mainly from the component with a frequency of $f_{1}=2780$ Hz and its higher-order harmonics, while only the fundamental component is retained in the filtered signal.

#### 2.3.2. Sound Pressure Calculation

Before carrying out exponential fitting, it was necessary to calculate the amplitude envelope function of the filtered noise signal. Accordingly, the signal was split into frames with a length of 0.2 ms, and the sound pressure amplitude of this segment was calculated frame by frame. The effective sound pressure was calculated as follows:

(2) $$pa=\sqrt{\frac{\sum {x_{i}}^{2}}{N}}$$

where *x_i_* is the amplitude of the audio signal and *N* is the number of data points contained in each frame. A smooth upper envelope signal was obtained after this step of processing.

#### 2.3.3. Determination of Growth Rate

The noise growth rate, characterizing the speed of development of the noise signal in a single acoustic emission event and reflecting the destabilizing tendency of the system, was calculated by an exponential fitting of the sound pressure signal [27]. For a given fitting interval, the corresponding noise growth rate can be obtained by

(3) $$\delta =\frac{1}{t_{ep}-t_{sp}}\ln\left(\frac{p_{e\_ep}}{p_{e\_sp}}\right)$$

where *t_sp_* and *t_ep_* represent the moments at which the start point (SP) and the end point (EP) of the fitted interval are located; *p_e_sp_* and *p_e_ep_* represent the effective values of sound pressure corresponding to the start and ending points, respectively.

Since the selection of fitting interval has a significant impact on the fitting results, the optimal growth rate was sought by varying the start and end points within the heuristic boundaries, which can be defined by taking the maximum effective sound pressure *p_e_max_* in a single acoustic emission event as a reference value. The effective sound pressure at the start and end points varied in the range of 0.1 to 0.2 and 0.7 to 0.85 times of *p_e_max_*, respectively, as shown in Figure 3f. Each pair of start and end points corresponded to a growth rate, and a growth rate set can be formed for a single acoustic emission event in turn. The fitting result was evaluated using the root mean square error (RMSE) to measure the fitting effect:

(4) $$\sigma =\sqrt{\frac{1}{N_{fit}}\sum_{1}^{N_{fit}}{\left(p_{e\_i}-p_{fit\_i}\right)}^{2}}$$

where *N_fit_* is the number of data points in the fitting interval; *p_e_* and *p_fit_* represent the filtered and fitted signals, respectively. Figure 3g shows the error values corresponding to the combinations of different start and end points, from which the fitting result with the smallest error was determined as the final growth rate.

## 3. Results and Discussion

### 3.1. Friction Characterization

**Figure 4:**
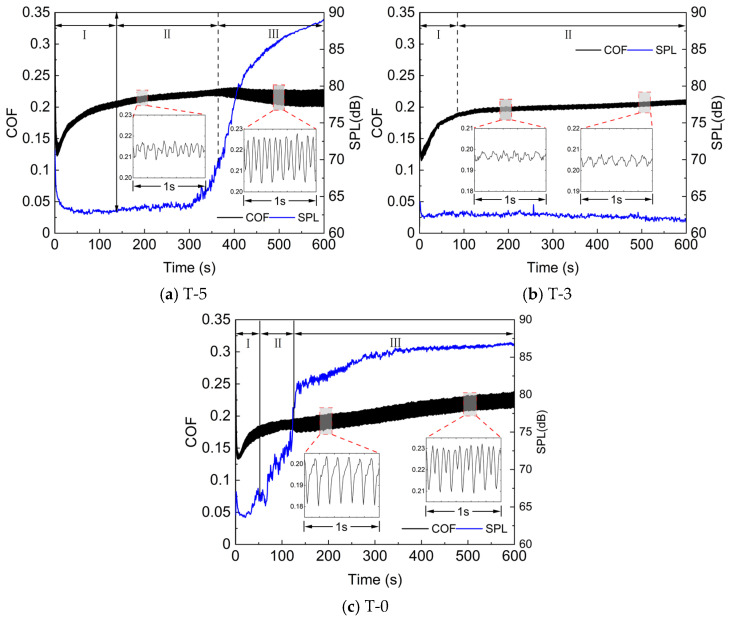
Instantaneous COF and SPL of each sample.

In this section, the friction behavior with different morphologies under long-duration sliding conditions is discussed, focusing on the evolution and development of instantaneous COF throughout the whole test period. Figure 4 illustrates the evolution of instantaneous COF and noise sound pressure level (SPL) of friction discs with different morphology characteristics.

From an overall point of view, the COFs of the three samples increased rapidly for a period of time before stabilizing; taking the fluctuation of the COF into account, the entire test process can be divided into three stages: the running-in stage, the stable wear stage, and the fluctuation stage. The instantaneous COF increases rapidly during the running-in stage, and then remains stable with little fluctuation, entering the stable wear stage. In the later stage of the test, the COF experiences increased fluctuation and enters a stage of violent fluctuation. However, the average value is still relatively stable, as shown by the widening curve in the graph.

Among the three samples, the stage division of the instantaneous COF of the T-5 sample is more typical and clearer. As shown in Figure 4a, the COF increased from 0.12 to about 0.21 in the first 140 s as a running-in stage, and then remained stable until 360 s when the fluctuation started to increase and entered into the fluctuation stage, in which the range of fluctuation expands from [0.21, 0.22] to [0.205, 0.225]. The T-3 sample only showed the first two stages in the full test cycle, and the COF remained stable around 0.195 after 85 s. In contrast, the T-0 sample did not exhibit an obvious steady wear stage, and the COF fluctuated significantly in the late running-in stage, with a further increase in fluctuations after 120 s. Among the three sets of tests, the sample T-3 had the longest duration of the stabilization phase, while the sample T-0 had the shortest.

### 3.2. Noise Characterization

Corresponding to the COF evolution, the development process of the noise signal can also be divided into three stages. The SPL of noise decreases gradually during the running-in stage and remains constant at a low noise level during the steady wear stage. However, during the fluctuation stage, the SPL rises rapidly.

As shown in Figure 4, during the running-in stage, the SPL of the T-5 sample decreased from 68 dB to 63 dB. After remaining at a low noise level for about 250 s, the SPL rapidly increased and eventually reached 89 dB. In contrast, the T-3 sample maintained a consistently low noise level throughout the test, with a maximum SPL of only 63 dB, which reflects the effective noise reduction properties of the texture feature. The noise signal of the T-0 sample shows two distinct slope changes at 30 s and 120 s, resulting in a rapid increase in SPL to over 80 dB at 130 s.

**Figure 5:**
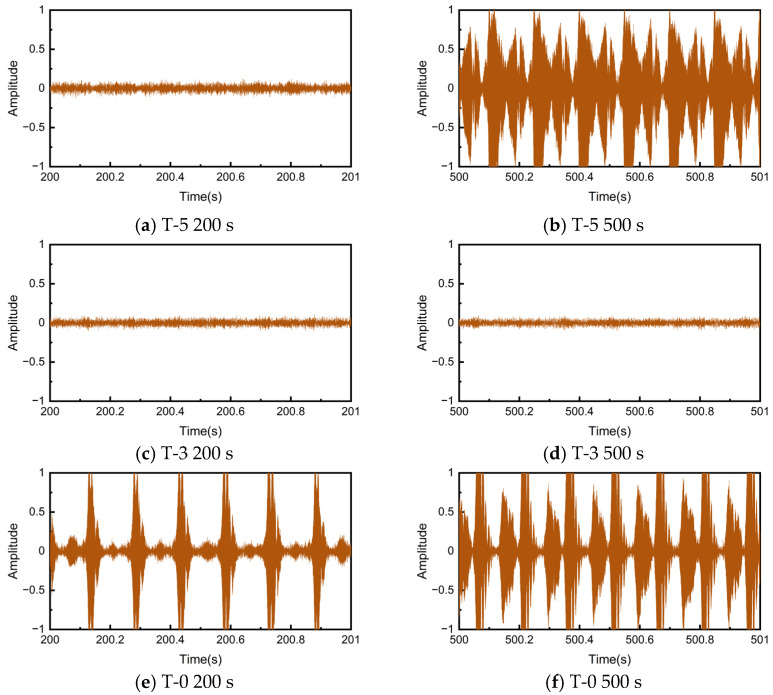
Short-time domain noise signals.

It is evident that there is a correlation between the instantaneous COF and the noise SPL change process, with a stronger noise signal tending to appear when the COF fluctuates drastically. As can be observed from the local magnification in Figure 4b, the COF of the T-3 sample is characterized by small amplitude and high frequency fluctuations, which corresponds to a lower SPL. Meanwhile, the COFs of the T-5 and T-0 samples exhibit large amplitude and low frequency oscillations highly correlated with the rotation speed. During the fluctuation stage, the COF of the T-5 sample experienced two sharp decreases in one rotation cycle, while the T-0 sample underwent an evolution from one decrease to two decreases in the COF during each cycle. Figure 5 illustrates the noise signals at the time corresponding to the local amplification in Figure 4. Although the two time series cannot be guaranteed to correspond exactly due to different signal acquisition devices, it can still be observed that the decrease times of the COF are consistent with the frequency of acoustic emission events.

### 3.3. Noise Growth Rate

The growth rates of single acoustic emission events at different time periods are compared for the T-5 and T-0 samples with significant noise.

**Figure 6:**
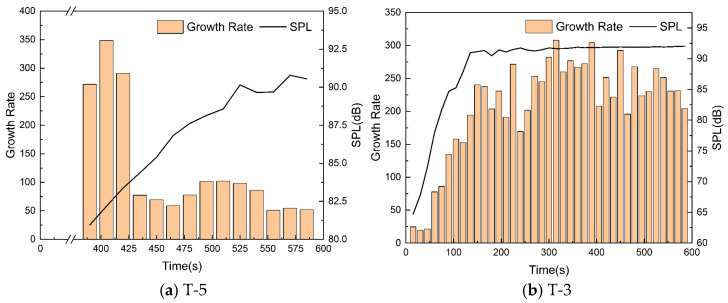
Noise growth rates and SPL of acoustic emission events.

Once the acoustic emission event was observed during the test, the acoustic emission noise signal was intercepted every 15 s, and its growth rate and SPL are calculated in Figure 6. It is important to note that the SPL here only represents the noise intensity of a single acoustic emission event and ignores the part with low-level noise between two times. Therefore, the calculated SPL is higher than that shown in Figure 4.

The sample T-5 had a high noise growth rate and low noise SPL at the onset of the squeal. The noise growth rate decreased and remained at a low level over time, while the SPL of the noise increased slowly. This suggests a low correlation between the two. However, there is a strong correlation between the noise growth rate and SPL for sample T-0. Both continued to increase until 135 s and stabilized at a high level afterwards.

The correlation difference between the two signals indicates two distinct noise development patterns. The noise from sample T-0 resembles a pulsed signal, characterized by a rapid increase in amplitude followed by a rapid decay with a shorter duration. As the sample wears out, the duration of each acoustic emission event remains constant. Therefore, an increase in the noise growth rate implies a larger maximum amplitude, and the final noise SPL is determined by the growth rate. The noise from sample T-5 resembles a sine wave signal, with a slower amplitude growth and decay and longer duration. As the sample wears out, the duration of each acoustic emission event gradually increases. Therefore, it is not possible to judge the noise intensity based solely on the growth rate, as even if the growth rate remains constant, the amplitude has more time to increase, resulting in a higher SPL.

### 3.4. Micro-Morphology Analysis

**Figure 7:**
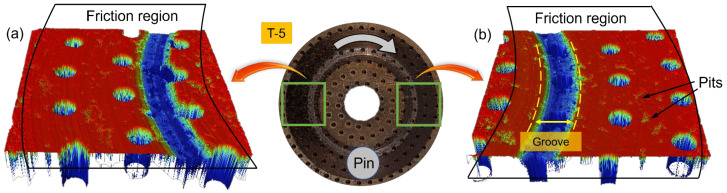

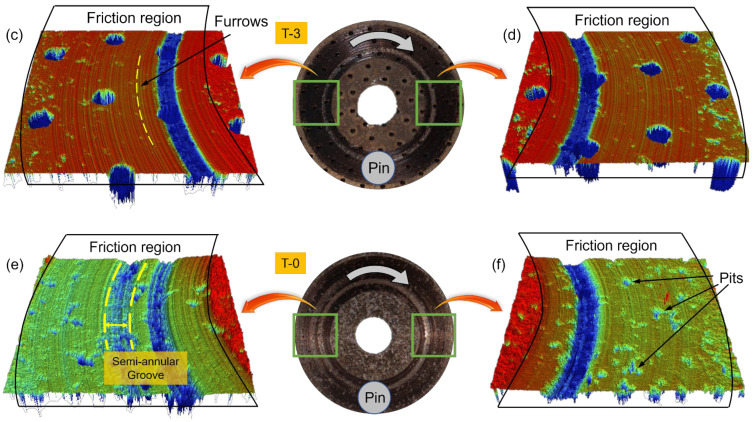
Micro-morphology of samples: (**a**) The morphology of the left area of the sample T-5; (**b**) The morphology of the right area of the sample T-5; (**c**) The morphology of the left area of the sample T-3; (**d**) The morphology of the right area of the sample T-3; (**e**) The morphology of the left area of the sample T-0; (**f**) The morphology of the right area of the sample T-0;.

The micro-morphology of two different friction areas after the test was scanned using a white light interferometer to obtain the wear condition of the sample. The scanning area was a square with a side length of 7 mm, and the scanning results are presented in Figure 7.

Deep circular grooves can be observed on the surfaces of the three samples after the test. Sample T-5 and T-3 display a single groove covering the surface of the circular texture. On the other hand, sample T-0 exhibits a complete groove on the inner side and a developing semi-annular groove on the outer side. The friction area beyond the deep grooves is relatively flat and constitutes the contact platform. Visible furrows can be observed on the contact platform of sample T-3, whereas sample T-5 and T-0 have shallower furrows and less surface roughness.

The morphology of sample T-3 in different regions of the same radius has good consistency from the circumferential direction. In addition to the semi-annular grooves, the sample T-0 also shows a large variation in the depth of wear of the intact groove, as evidenced by shallower depth in the area co-existing with the semi-annular groove, and vice versa. In Figure 7f, the groove depth is 46.5 μm, while in Figure 7e it is 27.8 μm. The circumferential variability of the sample T-5 is mainly observed in the region where the radius is smaller than the groove. In Figure 7a, the right side of the groove lacks texture units and exhibits furrow-like abrasion marks. In contrast, Figure 7b shows a large number of irregular micro-convex structures on the left side of the groove.

**Figure 8:**
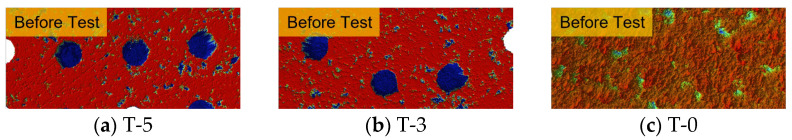

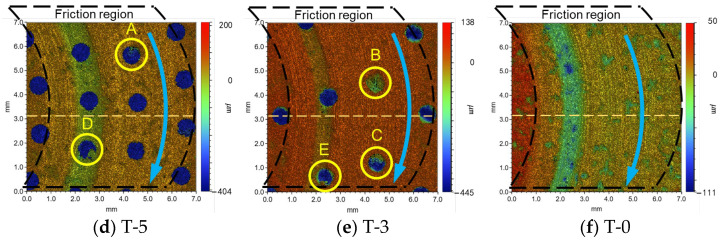
Two-dimensional morphology of wear area (The samples rotate in the direction of the blue arrow).

Figure 8 illustrates the 2D morphology of the samples. Abrasive debris is clearly visible in regions A, B, and C. The number of abrasive debris is significantly higher in sample T-3, with region B being almost completely filled with abrasive debris, possibly due to the lower density of the weaving units. The edges of the textures in areas D and E become irregular after experiencing heavy load friction, indicating that they are crushed and damaged under high contact pressure. In severe cases, the edges may even become partially detached.

**Figure 9:**
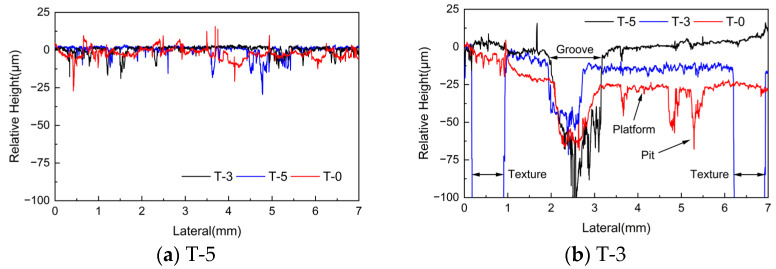
Relative height along the radial direction.

The left part of the scanned area did not experience friction and can be considered as the original height before the test. Taking this height as a reference, the relative height variation curve along the radial direction (i.e., the dashed part in Figure 8d–f) is extracted as shown in Figure 9. Both deep grooves and contact platforms can be observed in the wear areas of all three samples. Additionally, pits are found on the contact platform of sample T-0. The depth of wear on the contact platform decreases as the density of texture lines increases, and the relative heights of the contact platforms of samples T-5, T-3, and T-0 are −3 μm, −14 μm, and −28 μm, respectively. Conversely, the depth of the groove increases gradually, with the depths of 114.8 μm, 61.5 μm, and 46.5 μm of the three samples, respectively. The density of the texture affects the collection of abrasive debris, which in turn reduces wear caused by abrasive particles. However, increasing the texture density can also lead to increased wear in some areas due to the reduction in contact area and increase in local pressure. The roughness of the contact platforms of the three samples is significantly different due to the presence or absence of furrows, and the arithmetic mean deviation of the contours Ra in this region are 0.687 μm, 1.747 μm, and 0.942 μm, respectively.

Surface morphology has different effects on the friction noise at different stages of friction. During the running-in stage, the contact interface has more micro-convex peaks, resulting in a smaller actual contact area. This leads to localized stress concentrations that cause the micro-convex peaks to fracture, which is the source of the larger friction noise at the beginning of the test. At the same time, the abrasive particles thus created can change the form of friction from sliding friction to rolling friction, decreasing the COF. In the fluctuation stage, the intensification of wear makes the surface morphology of the friction disc to show a large difference in the circumferential direction, which causes periodic variation in the COF and leads to the instability of the system that emits high-intensity friction noise.

## 4. Relationship between Morphology and Noise

**Figure 10:**
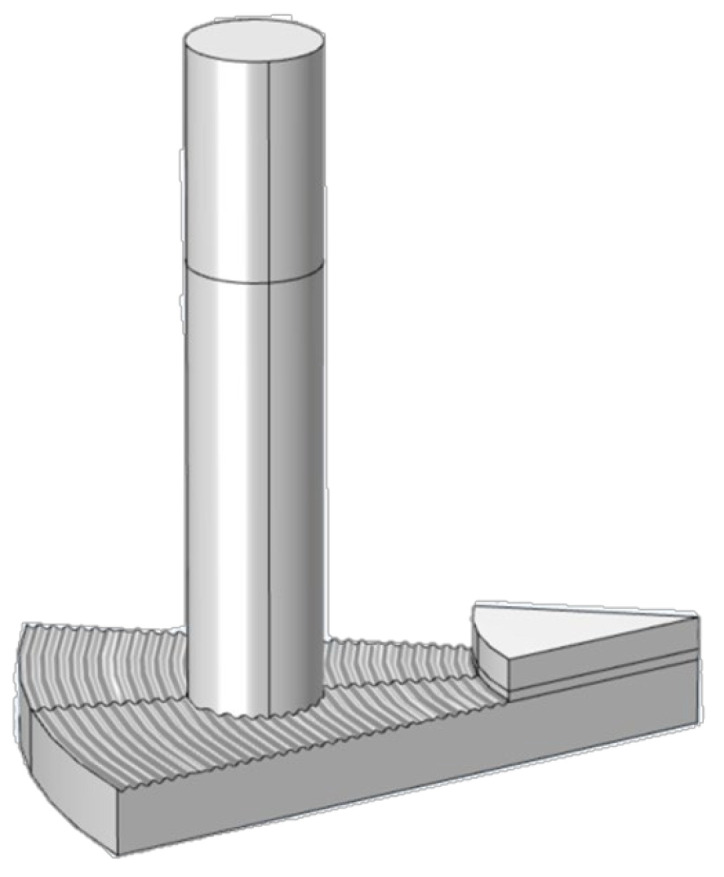
Finite element model.

Researches have demonstrated that the tendency of the system to generate high-frequency noise due to self-excited vibration is obviously enhanced when the stress is concentrated at the leading point of the contact interface [28,29]. To confirm the improvement of the stress distribution on the contact interface caused by the furrows of sample T-3, a finite element model, as shown in Figure 10, is established in COMSOL to analyze the changes in the surface pressure distribution. This model only considers the pin and the friction disc related to the contact state, where actual dimensions are used for the pin and the friction disc is modeled at 1/8. Taking the mesh density and the calculation cost into account, corrugation surface morphology is used to represent furrow wear marks, and the furrow features are amplified with a wavelength of 0.5 mm. Tetrahedral mesh is used in this model, with the maximum size of the mesh set to 0.004 mm on the contact interface. The pressure distribution of the different friction discs are calculated at 0 rpm and 400 rpm, respectively.

**Figure 11:**
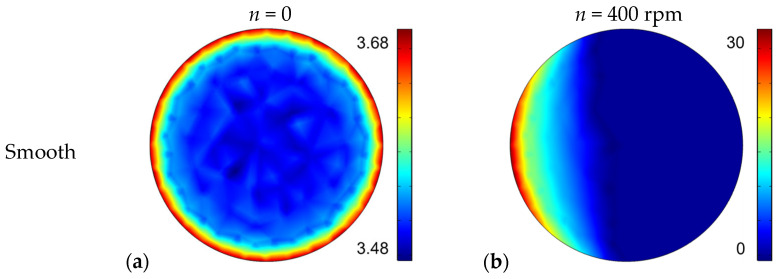

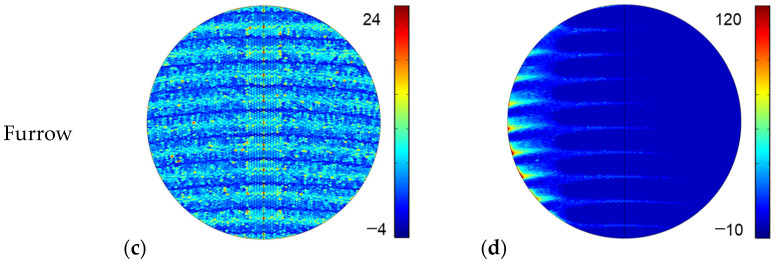
Contact pressure distribution on the pin surface: Contact pressure distribution of smooth sample at (**a**) 0 rpm and (**b**) 400 rpm; Contact pressure distribution of furrow sample at (**c**) 0 rpm and (**d**) 400 rpm.

The results of the stress distribution on the pin surface are shown in Figure 11. Stress concentrations at the pin leading point of the smooth sample indicate a strong tendency for self-excited vibration and squeal noise with high intensity and frequency (Figure 11a,b). The furrow-like surface has a larger maximum of local pressure, but its stress concentration area is more numerous and uniformly distributed (Figure 11c,d). The dispersion of high-pressure regions in the leading point can effectively reduce the instability tendency of the system and suppress the appearance of high-frequency noise.

## 5. Conclusions

This paper investigates the friction-wear and noise characteristics of the smooth sample and the circular pit texture samples with different line densities through pin-on-disc friction tests. The results were analyzed in conjunction with the surface morphology characteristics, and the following conclusions were obtained:

(1) Circular textures with varying parameters can extend the stable wear stage, delay the occurrence of sharp fluctuations in the COF, and reduce high-frequency noise. The most significant improvement was observed in the sample T-3.
(2) There is a correlation between the noise growth rate and the SPL in specific cases. Only when the duration of a single acoustic emission event is approximately constant can the noise growth rate determine the final noise intensity.
(3) The circular textures can collect abrasive debris and prevent the formation of the third body layer. Additionally, the surface may exhibit numerous furrow-like abrasion marks indicative of abrasive wear when the line density is appropriate (ρ = 0.3 for the purposes of this paper).
(4) The inclusion of furrows can enhance pressure distribution at the contact interface, decrease the instability tendency of the system, and prevent high-frequency squeal.

## Figures and Tables

**Table 1:** Material performance parameters.

Structures	Density (g/cm^3^)	Poisson’s Ratio	Modulus of Elasticity (GPa)	Thermal Conductivity (W/m/°C)
Pin and Core (65 Mn)	7.85	0.3	160	45.9
Lining (Cu-based)	5.5	0.3	6.2	9.3

**Table 2:** Test parameters.

Sample	Line Density ρ	Rotation Speed (rpm)	Load (N)	Radius (mm)
T-5	0.5	400	100	12
T-3	0.3	400	100	12
T-0	0	400	100	12

## Data Availability

The datasets presented in this article are not readily available because the data are part of an ongoing study. Requests to access the datasets should be directed to yuliang@bit.edu.cn.
